# Comparative chloroplast genomics reveals codon usage bias, simple sequence repeat dynamics, and phylogenomic relationships in Urticaceae

**DOI:** 10.3389/fpls.2026.1846589

**Published:** 2026-08-13

**Authors:** Fei Gao, Wendi Fan, Zishuo Wang, Guy Smagghe, Aneela Riaz, Ying Xue, Dong Li, Shushma Gosai, Yunpeng Gai

**Affiliations:** 1 School of Grassland Science, Beijing Forestry University, Beijing, China; 2 Department of Biology, Vrije Universiteit Brussel (VUB), Brussels, Belgium; 3 Institute of Entomology, Guizhou University, Guiyang, China; 4 Institute of Pure and Applied Biology, Bahauddin Zakariya University, Multan, Pakistan; 5 College of Natural Resource Management, Agriculture and Forestry University, Katari, Nepal

**Keywords:** codon usage bias, Ka/Ks analysis, phylogenomics, chloroplast genome, purifying selection, simple sequence repeats

## Abstract

Urticaceae is an ecologically diverse family, yet comparative studies on plastome structure and codon usage remain limited. To investigate the structural evolution and codon usage patterns in Urticaceae, we sequenced the complete chloroplast genome of *Urtica cannabina* and performed comparative analyses of plastome structure, codon usage bias, simple sequence repeats (SSRs), selective pressure, and phylogenetic relationships using plastome sequences from 48 Urticaceae species. The *Urtica cannabina* plastome (147,958 bp) displays a canonical quadripartite architecture, encoding a total of 128 genes (83 protein-coding, 37 tRNAs, and 8 rRNAs). Comparative analysis revealed a pronounced A/T bias (GC content: 36.56%), with a distinct decreasing GC gradient across codon positions (GC_1_ > GC_2_ > GC_3_). Relative synonymous codon usage (RSCU) analysis indicated a preference for A/U-ending codons, and the average effective number of codons (ENC) values ranged from 45.59 to 47.22, indicating generally weak codon bias. Parity rule 2 (PR2) analysis indicated a consistent preference for G and T at the third codon position in 48 Urticaceae chloroplast genomes, with highly conserved base usage across all species. Furthermore, a total of 3,552 SSRs were identified family-wide, with mononucleotide repeats (71.85%) predominating in intergenic regions. Ka/Ks analysis indicated that 97.1% of protein-coding genes were under purifying selection (Ka/Ks < 0.5). Phylogenetic analysis showed that *Hesperocnide tenella* is closely related to the genus *Urtica*. This study characterizes the structural features and codon usage patterns of chloroplast genomes in Urticaceae, providing new insights into their evolutionary dynamics and establishing a basis for investigating the interplay among genomic traits, codon bias, and adaptive evolution.

## 1 Introduction

The family Urticaceae comprises approximately 54 genera and 2,625 species, representing a significant component of the global temperate and tropical flora (Christenhusz and Byng, 2016). Members of this family occupy a wide range of ecological niches and play important roles in ecosystem functioning, including soil stabilization and the provision of food and shelter for diverse animal communities (Parente et al., 2024). Beyond their ecological roles, several Urticaceae species are of significant economic and pharmacological value. For instance, *Urtica dioica* is a cornerstone of both traditional and modern medicine, possessing documented anti-inflammatory, antihistamine, and antioxidant properties with therapeutic potential for autoimmune and dermatological conditions (Devkota et al., 2022; Khare et al., 2012). Furthermore, fiber crops such as *Boehmeria nivea* remain industrial staples globally (Maietti et al., 2021; Viotti et al., 2024). Known for their diverse ecological adaptations and specialized morphological features—such as stinging hairs and specialized explosive dehiscent stamens—members of this family, particularly *Urtica*, are of paramount importance in ethnobotany and pharmacology (Li et al., 2022). The broad geographic distribution, ecological versatility, and economic value of Urticaceae make this family an informative model for studying plant evolution, adaptation, and genome evolution (Viotti et al., 2022). Despite this importance, the plastome-level evolutionary patterns associated with ecological diversification in Urticaceae remain insufficiently investigated.

Chloroplasts are essential plant organelles responsible for photosynthesis and central metabolic processes and originated from cyanobacteria (Daniell et al., 2016). Most land plant chloroplasts retain a complete, circular genome typically organized into a conserved quadripartite structure composed of a large single-copy (LSC) region, a small single-copy (SSC) region, and two inverted repeat (IR) regions (Kwasniak-Owczarek et al., 2024). Due to their generally uniparental, predominantly maternal inheritance, relatively small genome size, limited recombination, and moderate nucleotide substitution rates, chloroplast genomes have become powerful molecular resources for plant systematics, phylogenetics, and population genetic studies (Agarwal et al., 2008). Compared with nuclear and mitochondrial genomes, chloroplast genomes exhibit a high degree of structural conservation, making them useful for resolving evolutionary relationships at multiple taxonomic levels. Beyond phylogenetic inference, chloroplast genomes also enable investigations into genome organization, sequence divergence, and patterns of molecular evolution across lineages. High-throughput sequencing now enables rapid plastome characterization, providing a robust framework for resolving evolutionary relationships in taxa with complex histories. While this approach has been successfully applied to families such as Asteraceae and Poaceae (Mandel et al., 2019; Soreng et al., 2017; Wysocki et al., 2015), its application within the Urticaceae remains fragmented, particularly regarding lineages with ambiguous taxonomic boundaries.

An important aspect of chloroplast genome evolution is codon usage bias (CUB), defined as the non-random use of synonymous codons encoding the same amino acid. Codon usage patterns are shaped by a combination of evolutionary forces, including mutational pressure, natural selection, and genetic drift (Chen et al., 2024). Due to the degeneracy of the genetic code, nucleotide variation at the third codon position often does not alter the encoded amino acid, making this position particularly informative for examining the relative contributions of mutational biases and selective constraints. CUB can influence translational efficiency and accuracy and may affect protein expression levels, fitness, and environmental adaptation (Alshegaihi et al., 2023). In chloroplast genomes, a pronounced bias toward A/T-rich codons has been reported in many angiosperm lineages. However, the extent to which this bias reflects neutral mutational processes versus adaptive selection related to photosynthetic function, gene expression, or ecological specialization remains an open biological question. Analyses of GC content variation across different codon positions can further illuminate differential selective pressures acting on chloroplast genes (Guan et al., 2018; Zhang and Feng, 2025).

Previous studies of Urticaceae plastomes have largely focused on structural characterization and broad phylogenetic placement (Huang et al., 2019; Li et al., 2021; Ogoma et al., 2022). Comparatively little attention has been paid to codon usage evolution, selective pressures acting on chloroplast genes, and the potential adaptive significance of structural variation. Determining whether these genomic patterns are driven by neutral mutational processes or functional selection is essential for both evolutionary theory and the development of molecular markers for genetic improvement (He et al., 2021; Rasheed et al., 2022).

In this study, we sequenced and assembled the complete chloroplast genome of *Urtica cannabina* and performed a comparative analysis involving 48 Urticaceae species. Our primary objective was to resolve the phylogenetic relationships within Urticaceae at higher resolution. To systematically explore evolutionary characteristics, we employed a suite of comparative analyses, including whole-genome collinearity assessment, IR boundary shift analysis, simple sequence repeat (SSR) identification, and phylogenetic reconstruction. For phylogenetic reconstruction, we utilized maximum-likelihood methods to construct trees based on complete chloroplast genome sequences (Shi et al., 2024). We further aimed to provide a high-resolution phylogenomic reconstruction to resolve contentious relationships.

## 2 Materials and methods

### 2.1 Sample collection, DNA extraction, and genome sequencing

Fresh young leaves of *U. cannabina* were collected from Yinchuan, Ningxia, China (38.47°N, 106.20°E), and samples were immediately flash-frozen in liquid nitrogen and stored at -80°C to maintain DNA integrity. This species was selected based on previous phylogenetic analyses that identified it as a key representative of the family. Total genomic DNA was extracted using a modified cetyl trimethyl ammonium bromide (CTAB) method (Carey et al., 2023). Chloroplast genomes were sequenced by Beijing BioMarker Biotechnology using the Illumina NovaSeq platform. For comparative analysis, the chloroplast DNA sequences of additional Urticaceae taxa and Moraceae outgroups were obtained from the National Center for Biotechnology Information (NCBI) database (https://www.ncbi.nlm.nih.gov/nuccore/). These sequences were used to reconstruct phylogenetic relationships across Urticaceae. The complete chloroplast genome sequence of *U. cannabina* was deposited in the NCBI GenBank database under accession number PQ846489 on January 6, 2025.

### 2.2 Chloroplast genome assembly and annotation

The chloroplast genome of *U. cannabina* was assembled using NOVOPlasty v4.3.3 in chloroplast mode (Dierckxsens et al., 2017). The Illumina dataset consisted of paired-end reads with a read length of 150 bp and an initial insert size setting of 263 bp. The platform was specified as Illumina, and the library mode was set to paired-end. Hash storage, automatic insert-size estimation, and quality-score usage were enabled, while ambiguous N bases were reduced during assembly. High-resolution circular genome map was generated using Chloroplot (Zheng et al., 2020). The obtained circular plastome sequences were annotated using GeSeq on the Chlorobox platform (https://chlorobox.mpimp-golm.mpg.de/geseq.html). To analyze structural evolution, the expansion and contraction of inverted repeat (IR) boundaries were visualized and compared across Urticaceae species using IRscope (irscope.shinyapps.io/irapp/) (Amiryousefi et al., 2018).

### 2.3 Sequencing-depth validation and coverage visualization

To evaluate the reliability and uniformity of the chloroplast genome assembly, paired-end reads were mapped back to the assembled plastome. Read alignment was performed using BWA-MEM, followed by SAM/BAM conversion, sorting, and indexing with SAMtools (Li et al., 2009). Per-position sequencing depth was extracted using the SAMtools depth command, generating a file containing the chromosome, position, and depth at each covered site. Based on this file, we computed coverage statistics, including coverage rate, depth distribution (mean, median, quartiles, maximum, SD, CV), and uniformity. All analyses were performed in R v4.2.3 using the packages ggplot2, dplyr, scales, patchwork, and openxlsx.

### 2.4 Phylogenetic analysis

Maximum-likelihood phylogeny was inferred from concatenated plastome protein-coding sequences of 111 species representing 30 genera within Urticaceae and the outgroup family Moraceae. Each protein-coding gene was aligned separately using MAFFT v7.505, and ambiguously aligned regions were removed using trimAl v1.4 (Capella-Gutierrez et al., 2009; Katoh and Standley, 2013). The curated alignments were concatenated into a supermatrix for subsequent phylogenetic analyses. Maximum likelihood (ML) phylogenetic analyses were performed using IQ-TREE2 v2.2.0 (Minh et al., 2020). The best-fit nucleotide substitution model was selected with ModelFinder, as implemented in IQ-TREE v2.2.0, according to the Bayesian Information Criterion (BIC) (Kalyaanamoorthy et al., 2017). Nodal support was evaluated using ultrafast bootstrap approximation (UFBoot2; 1,000 replicates), Shimodaira–Hasegawa approximate likelihood ratio tests (SH-aLRT; 1,000 replicates), and the approximate Bayes test (aBayes) (Anisimova et al., 2011; Hoang et al., 2018).

### 2.5 Ancestral state reconstruction

Ancestral state reconstruction for continuous chloroplast genome traits was performed using the phytools v2.0 package in R v4.2.3 (Revell, 2024). A Brownian motion model of trait evolution was assumed, and maximum likelihood estimation was used to infer ancestral values at internal nodes. Ancestral states were estimated using the fastAnc function, which employs a rerooting algorithm for computational efficiency. The reconstructed values were visualized by mapping continuous trait variation onto the maximum likelihood (ML) phylogeny using the contMap function. These specific traits were selected based on their established relevance for characterizing plastome diversity and evolutionary patterns within Urticaceae (Wang et al., 2020; Zhan et al., 2024). Ancestral character state reconstruction was performed for eight continuous chloroplast genome traits: (1) evolutionary rate, (2) GC content (%), (3) genome size (kb), (4) gene density, (5) coding ratio (%), (6) number of tRNA genes, (7) inverted repeat (IR) length (kb), and (8) AT skew.

### 2.6 Statistical analysis of chloroplast genome variations

Chloroplast genome characteristics were statistically compared across 11 genera of Urticaceae. *Urtica* exhibited the largest sampling coverage with 20 species, followed by *Pilea* (17 species) and *Laportea* (10 species). Three genera possessed moderate sample sizes ranging from 5 to 9 species, while the remaining five genera each contained four species with balanced representation. Data distribution and homogeneity of variances were evaluated using the Shapiro-Wilk and Levene’s test, respectively. As the assumptions of normality were not consistently met, non-parametric methods were applied. Significant differences among multiple groups were tested using the Kruskal-Wallis test. When significant differences were detected (α = 0.05), post hoc pairwise comparisons were conducted using Dunn’s test with Holm correction to account for multiple testing. Effect sizes were reported as epsilon-squared (ϵ²) for Kruskal-Wallis tests. Effect sizes were calculated using ϵ² and interpreted as small (0.01–0.05), medium (0.06–0.13), and large (≥0.14). All statistical analyses were conducted in R v4.2.3 using the packages rstatix, dunn.test, car, phytools, effectsize, and related packages. Data visualization was performed using ggplot2, ggbeeswarm, and patchwork to generate violin plots overlaid with boxplots and individual data points.

### 2.7 Collinearity analysis

To assess structural conservation and identify homologous regions across the Urticaceae, we performed a family-wide collinearity analysis using 48 complete plastomes retrieved from the GenBank database. Gene features were extracted using BioPerl v1.7 and converted into GFF3 format (Stajich, 2007). Pairwise whole-genome alignments were performed with NUCmer in the MUMmer package v4.0 using parameters “-c 200 -g 200”. Alignment outputs were used to estimate nucleotide identity (ANI) values and comparative coverage metrics (Yoon et al., 2017). In addition, BLASTn was used to identify syntenic regions across genomes. Collinearity relationships were visualized using the genoPlotR package in R, where gene fragments were color-coded by functional category and similarity blocks were shaded according to nucleotide identity (Guy et al., 2010). Average nucleotide identity (ANI) values were calculated separately and visualized as heatmaps using the ComplexHeatmap package (Gu et al., 2016). Hierarchical clustering based on the ANI distance matrix was performed using the Ward.D2 method and visualized with functions from the ggtree and ape packages (Paradis and Schliep, 2019; Yu et al., 2016).

### 2.8 Simple sequence repeat analysis

SSRs are highly polymorphic genetic markers composed of tandem repeats of short nucleotide motifs, and are widely used in population genetics, species identification, and genetic diversity studies (Lewis et al., 2020). To investigate SSR composition and distribution, the complete chloroplast genomes of *U. cannabina* and 47 additional Urticaceae species were systematically analyzed. SSR detection was performed using the MISA tool (https://webblast.ipk-gatersleben.de/misa/) (Beier et al., 2017), with minimum repeat thresholds of 10, 5, 4, 3, 3, 3, 3, 3, 3, and 3 for mono-, di-, tri-, tetra-, penta-, hexa-, hepta-, octa-, nona-, and decanucleotide motifs, respectively. Detected SSRs were categorized according to motif type and genomic location. Visualization of SSR distribution was carried out using the ggplot2 package v3.4.0 in R to illustrate interspecific variation in SSR frequency and composition across Urticaceae chloroplast genomes.

### 2.9 Relative synonymous codon usage analysis

Relative synonymous codon usage (RSCU) was calculated to quantify CUB by comparing the observed frequency of each synonymous codon with its expected frequency under equal usage assumptions (Khandia et al., 2024; Sharp et al., 1986). Protein-coding gene sequences from the chloroplast genomes were extracted using PhyloSuite. RSCU values were calculated with CodonW v1.4.4 (https://codonw.sourceforge.net/). To visualize codon usage patterns across species, a heatmap of RSCU values was generated using the pheatmap package in R. An RSCU value > 1 indicates preferential codon usage, = 1 indicates no bias, and < 1 indicates underrepresentation. These analyses were used to assess nucleotide composition preferences and to explore the evolutionary forces shaping codon usage patterns in Urticaceae chloroplast genomes.

### 2.10 Effective number of codons analysis

The effective number of codons (ENC) was used to quantify codon usage bias, with values ranging from 20 (maximum bias; only one codon used per amino acid) to 61 (no bias) (Sun et al., 2012). ENC plot analysis was performed by plotting ENC values against GC content at the third codon position (GC3_S_) for each gene. A theoretical curve representing the expected relationship between ENC and GC3_S_ under neutral mutational pressure was superimposed. The distribution of genes relative to this curve was used to infer the underlying forces shaping codon usage: genes located near the curve are primarily influenced by mutational pressure, whereas those deviating substantially are considered to be affected by natural selection or other evolutionary factors (Fuglsang, 2006).

### 2.11 Parity rule 2 analysis

Parity rule 2 (PR2) analysis was conducted to evaluate nucleotide usage bias at the third codon position and to further disentangle the relative contribution of mutation and selection to codon usage patterns (Pflughaupt and Sahakyan, 2023). The ratios G_3_/(G_3_+C_3_) and A_3_/(A_3_+T_3_) were calculated for each gene, where G_3_, A_3_, C_3_, and T_3_ represent the frequencies of guanine, adenine, cytosine, and thymine at the third codon position, respectively. These values were plotted on the PR2 scatter plot, with G_3_/(G_3_+C_3_) on the x-axis and A_3_/(A_3_+T_3_) on the y-axis. The central point (0.5, 0.5) represents equal usage of complementary bases (no bias), while deviations from this point reflect asymmetric nucleotide usage resulting from the combined effects of mutational bias and natural selection (Yang et al., 2024).

### 2.12 Neutrality analysis

Neutrality analysis was conducted to evaluate the selective pressures acting on codon usage within the chloroplast genomes of Urticaceae (Wang et al., 2018). This analysis employed the GC content at the first and second (GC_12_) and third (GC_3_) codon positions, which are subject to distinct evolutionary forces. A scatter plot was constructed with GC_12_ on the y-axis and GC_3_ on the x-axis, with each gene positioned within this coordinate system. The slope of the regression line (k) indicates the relative influence of mutation versus selection: if k ≈ 1, all points fall along the diagonal, indicating GC_12_ and GC_3_ are highly correlated, and the codon usage bias is mainly driven by mutational pressure; if k ≈ 0, selection is inferred to make a greater relative contribution (Nasrullah et al., 2015).

### 2.13 Nucleotide diversity analysis

Nucleotide diversity (π) was calculated following Nei and Li (Nei and Li, 1979), representing the average number of pairwise nucleotide differences per site among sequences. Annotated coding sequences (CDSs) were parsed from 48 Urticaceae chloroplast GenBank records, with *Urtica cannabina* used as the reference for gene order and regional assignment. Orthologous CDS groups were identified by gene annotation, and duplicated inverted-repeat genes were collapsed to one representative copy. Trans-spliced and compound-location CDSs were handled using annotation-aware extraction and quality-control checks. Multiple sequence alignments for each gene were generated using MAFFT v7.505 (with the --auto option). Poorly aligned regions were removed using trimAl v1.4 with the -automated1 option.

### 2.14 Selection pressure analysis

Selection pressure acting on chloroplast protein-coding genes was evaluated by comparing nonsynonymous (Ka) and synonymous (Ks) substitution rates (Zhang et al., 2025). For each orthogroup, CDSs were first checked for reading-frame consistency, ambiguous codons, and internal stop codons. Terminal stop codons were removed before codon alignment. To avoid frame-disrupting indels from direct nucleotide alignment, the workflow generated protein-guided codon alignments: CDSs were translated into amino-acid sequences, the protein sequences were aligned with MAFFT, and the aligned amino-acid columns were back-translated into codon triplets. The Ka/Ks ratio was calculated using KaKs_Calculator v2.0 (https://sourceforge.net/projects/kakscalculator2/) with the Nei-Gojobori method (Wang et al., 2010). The Ka/Ks ratio was interpreted as follows: values > 1 indicate positive selection, values ≈ 1 suggest neutral evolution, and values < 1 indicate purifying selection, reflecting the removal of deleterious nonsynonymous mutations (Yang and Nielsen, 2000).

## 3 Results

### 3.1 Basic characteristics of the chloroplast genomes

**Figure 1:**
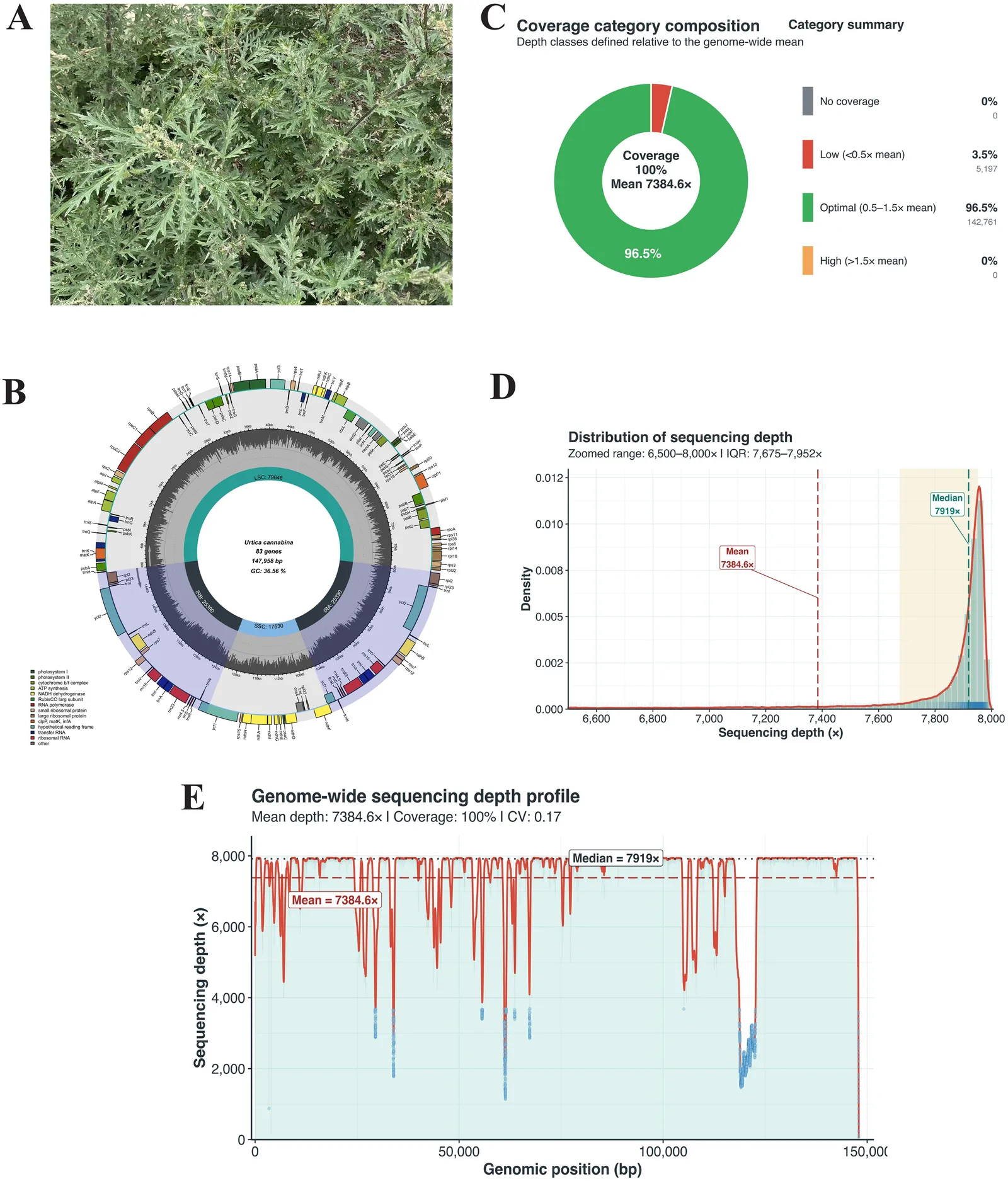
Morphology, chloroplast genome organization, and sequencing-depth validation of *Urtica cannabina*. **(A)** Photograph of *U. cannabina*. **(B)** Circular map of the *U. cannabina* chloroplast genome generated using Chloroplot. Genes are color-coded by functional category and are shown according to their orientation on the plastome. The inner tracks indicate GC content variation and the positions of the LSC, SSC, IRa, and IRb regions. **(C)** Coverage category composition based on deviation from the genome-wide mean sequencing depth. **(D)** Zoomed distribution of sequencing depth across the main depth range, with the interquartile range and mean/median values indicated. **(E)** Genome-wide sequencing-depth profile of the assembled chloroplast genome. The dashed and dotted horizontal lines indicate the mean and median depths, respectively.

The complete chloroplast genome of *U. cannabina* (**Figure 1A**) exhibits a typical quadripartite structure, comprising a large single-copy (LSC) region (79,648 bp), a small single-copy (SSC) region (17,530 bp) and two inverted repeat (IR) regions (25,390 bp) (**Figure 1B**). The total genome length of *U. cannabina* is 147,958 bp, with an overall GC content of 36.56% (**Table 1**). This structural organization is consistent with most land plant chloroplast genomes, particularly within the Urticaceae. The genome contains a total of 128 genes, including 83 protein-coding genes, 37 tRNA genes, and 8 rRNA genes. Read-mapping validation showed that the assembled *U. cannabina* chloroplast genome was fully covered by Illumina reads. All 147,958 positions in the plastome had non-zero sequencing depth, corresponding to a coverage rate of 100.0%. The genome-wide mean depth was 7,384.6×, while the median depth was 7,919×, indicating that most genomic positions were covered at very high depth (**Figure 1E**). The first and third quartiles were 7,675× and 7,952×, and the maximum observed depth was 8,000×. The coefficient of variation in sequencing depth was 0.17, and the coverage uniformity index was 0.83 (**Figure 1D**). Coverage classification further confirmed that the vast majority of positions fell within the expected depth range. Among all plastome positions, 142,761 positions (96.5%) were classified as optimal coverage, defined as 0.5–1.5× the genome-wide mean depth. A smaller fraction of the genome, comprising 5,197 positions (3.5%), was classified as low coverage, whereas no positions were assigned to the no-coverage or high-coverage classes (**Figure 1C**). This pattern indicates that the assembly was supported across the entire reference length and that extremely high-depth outlier regions were not detected under the applied classification scheme.

**Table 1:** Genomic features and nucleotide composition of 48 Urticaceae chloroplast genomes.

GenBank accession number	Species Name	Total length (bp)	Total GC (%)	GC1%	GC2%	GC3%	GC3s	Protein-coding gene num	rRNA gene num	tRNA gene num	Total gene num	Average ENC
NC_039763	*Cecropia pachystachya*	153925	36.55	45.3	37.69	29.22	26.2	84	8	37	129	46.224
NC_041413	*Debregeasia orientalis*	160283	36.13	44.79	37.37	29.26	26.27	83	8	37	128	46.544
NC_047192	*Elatostema dissectum*	150302	36.23	45.1	37.41	28.47	25.55	86	8	36	130	45.585
NC_054339	*Pilea peperomioides*	152327	36.35	44.82	37.42	29.21	26.21	86	8	37	131	46.749
NC_054341	*Pilea mollis*	150587	36.72	45	37.5	29.39	26.39	86	8	37	131	47.009
NC_054344	*Pilea microphylla*	150019	36.61	44.66	37.59	29.28	26.26	87	8	37	132	46.741
NC_054345	*Pilea pumila*	152036	36.24	44.93	37.42	29.11	26.13	87	8	37	132	46.587
NC_056276	*Procris crenata*	154124	36.53	45.25	37.65	29.25	26.3	86	8	37	131	45.793
NC_056894	*Boehmeria nivea*	156065	36.33	45.04	37.56	29.25	26.3	84	8	37	129	46.171
NC_056983	*Poikilospermum lanceolatum*	153454	36.89	45.4	37.74	29.71	26.73	86	8	37	131	46.936
NC_061041	*Elatostema stewardii*	150263	36.35	45.02	37.47	28.61	25.65	85	8	36	129	46.242
NC_061695	*Pellionia scabra*	153220	36.42	44.92	37.44	29.17	26.15	85	8	37	130	45.727
NC_062307	*Parietaria floridana*	153538	36.21	45.15	37.66	28.74	25.81	83	8	37	128	45.968
NC_062308	*Gesnouinia arborea*	153065	36.32	45.01	37.61	28.68	25.75	83	8	37	128	45.91
NC_062309	*Forsskaolea angustifolia*	156714	36.15	45.01	37.53	29.24	26.26	83	8	37	128	46.592
NC_062310	*Didymodoxa caffra*	154684	36.37	44.89	37.31	29.31	26.32	81	8	37	126	46.603
NC_062312	*Boehmeria japonica*	156266	35.66	44.62	37.1	28.25	25.3	85	8	37	130	45.736
NC_062313	*Oreocnide trinervis*	156793	36.32	45.21	37.65	29.44	26.48	84	8	37	129	47.065
NC_062318	*Oreocnide pedunculata*	157046	36.21	45.17	37.61	29.33	26.36	84	8	37	129	46.942
NC_063549	*Debregeasia elliptica*	155901	36.3	44.75	37.39	29.27	26.28	83	8	37	128	46.632
NC_064736	*Australina pusilla*	152624	36.51	45.05	37.46	29.45	26.45	84	8	37	129	47.014
NC_064738	*Dendrocnide meyeniana*	152621	36.65	45.11	37.65	29.32	26.31	84	8	37	129	46.205
NC_064739	*Dendrocnide sinuata*	152650	36.61	45.04	37.53	29.38	26.37	85	8	37	130	46.203
NC_064742	*Zhengyia shennongensis*	150109	36.83	45.24	37.71	29.18	26.21	84	8	37	129	46.508
NC_064743	*Gyrotaenia microcarpa*	153911	36.55	45.19	37.51	29.39	26.36	85	8	37	130	45.902
NC_064744	*Gyrotaenia myriocarpa*	153979	36.53	45.15	37.5	29.33	26.31	85	8	37	130	45.918
NC_064745	*Hesperocnide tenella*	146864	36.37	44.74	37.47	28.74	25.74	83	8	37	128	46.348
NC_064747	*Laportea canadensis*	150253	36.8	45.26	37.81	29.29	26.3	84	8	37	129	46.708
NC_064748	*Laportea cuspidata*	149149	36.93	45.12	37.74	29.09	26.11	83	8	37	128	46.075
NC_064751	*Leucosyke puya*	156511	35.99	44.83	37.49	29.04	26.02	83	8	37	128	45.991
NC_064934	*Nanocnide japonica*	145970	36.64	45	37.51	28.43	25.45	82	8	37	127	45.635
NC_064935	*Nanocnide lobata*	145302	36.63	44.82	37.33	28.61	25.62	83	8	37	128	45.744
NC_064936	*Obetia aldabrensis*	153239	36.79	45.48	37.78	29.76	26.77	84	8	37	129	46.886
NC_064937	*Poikilospermum cordifolium*	153801	36.84	45.48	37.81	29.72	26.74	84	8	37	129	46.647
NC_064938	*Rousselia humilis*	153304	36.03	44.6	37.29	28.73	25.71	85	8	37	130	46.093
NC_064939	*Touchardia latifolia*	152871	36.64	45.38	37.57	29.54	26.55	84	8	37	129	46.297
NC_064940	*Urera baccifera*	153215	36.69	45.34	37.8	29.59	26.6	84	8	37	129	46.63
NC_064944	*Urtica angustifolia*	146703	36.62	44.87	37.52	29.03	26.04	83	8	37	128	46.688
NC_064948	*Urtica dioica*	147799	36.57	44.66	37.36	29.08	26.1	83	8	37	128	47.053
NC_064950	*Urtica hyperborea*	147898	36.55	44.79	37.48	28.88	25.86	82	8	37	127	46.812
NC_064952	*Urtica magellanica*	146606	36.54	44.96	37.52	29.08	26.07	83	8	37	128	46.557
NC_064958	*Urtica urens*	147544	36.33	44.51	37.32	28.66	25.7	84	8	37	129	47.099
NC_064969	*Girardinia diversifolia*	152818	36.96	45.26	37.78	29.55	26.58	85	8	37	130	47.218
NC_064974	*Urera cameroonensis*	153212	36.88	45.49	37.85	29.75	26.75	84	8	37	129	46.484
NC_064977	*Laportea decumana*	151855	36.74	45.08	37.65	29.36	26.37	84	8	37	129	46.519
NC_064980	*Girardinia bullosa*	152388	36.95	45.42	37.81	29.6	26.62	84	8	37	129	46.466
NC_066035	*Parietaria judaica*	152689	36.29	45.02	37.48	28.73	25.78	85	8	37	130	46.024
PQ846489	*Urtica cannabina*	147958	36.56	44.65	37.38	29.03	26.04	83	8	37	128	46.928

### 3.2 Nucleotide composition analysis of Urticaceae chloroplast genomes

Analysis of nucleotide composition across 48 Urticaceae chloroplast genomes revealed a pronounced bias toward adenine (A) and thymine (T). The average nucleotide frequencies were 31.33% adenine (A), 32.17% thymine (T), 18.56% cytosine (C), and 17.94% guanine (G) (**Supplementary Table 1**), resulting in substantially higher A + T content than C + G. Overall GC content varied only slightly among species, ranging from 35.66% to 36.96%, indicating strong compositional conservation within the family. Analysis of GC content across codon positions revealed a clear decreasing trend, with mean values of 45.03% (GC_1)_, 37.55% (GC_2)_, and 29.16% (GC_3_). The markedly lower GC content at the third codon position indicates a strong preference for A- or T-ending synonymous codons. The low GC_3_ value (29.16%) further highlights strong AT enrichment at synonymous sites, which may be associated with translational efficiency or gene expression regulation. From a structural perspective, higher AT content may also influence DNA flexibility and stability, as A-T base pairs form two hydrogen bonds compared with three in G–C pairs. Across the 48 species analyzed, average ENC values ranged from 45.59 to 47.22, indicating relatively weak overall CUB. These values suggest weak codon bias rather than strong specialization, consistent with the conserved nature of plastid gene expression systems.

### 3.3 Phylogenetic analysis

**Figure 2:**
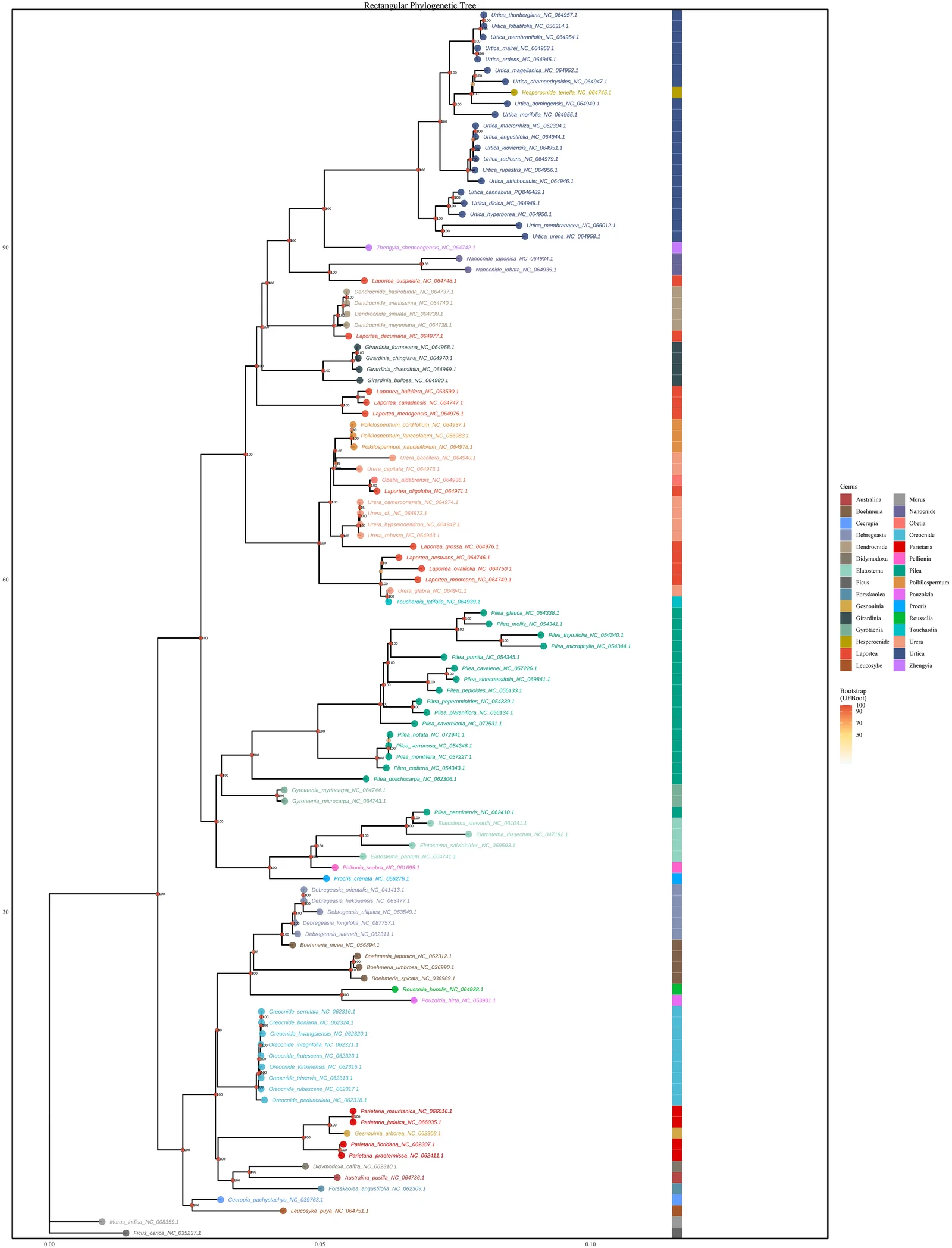
Rectangular phylogenetic tree of Urticaceae species with genus-level classification. Maximum-likelihood phylogeny inferred from concatenated plastome protein-coding sequences of 111 species representing 30 genera within Urticaceae and the outgroup family Moraceae. The ingroup includes representatives from the tribes Urticeae, Boehmerieae, Elatostemateae, Forsskaoleeae, Sarcochlamydeae, Cecropieae and Parietarieae. Colored tip points indicate genus assignments. The vertical color strip shows genus groupings. UFBoot/bootstrap values > 70% are shown at nodes.

An expanded maximum-likelihood phylogeny was conducted based on concatenated chloroplast protein-coding sequences derived from the complete chloroplast genomes of *U. cannabina* and other Urticaceae species, with Moraceae taxa (*Morus indica, Ficus carica*) serving as outgroups. The two Moraceae outgroups were clearly separated from the Urticaceae ingroup, supporting the appropriateness of the rooted tree topology for family-level interpretation. Most internal nodes within Urticaceae were strongly supported (UFBoot ≥ 95%; SH-aLRT ≥ 80%), highlighting the strong phylogenetic signal in the concatenated chloroplast protein-coding supermatrix (**Figure 2**). *Urtica* species formed a compact and well-supported lineage with comparatively short internal branches. *U. cannabina* clustered closely with *U. dioica* and *Urtica hyperborea*, forming a well-supported clade. *Hesperocnide tenella* exhibited a close phylogenetic relationship with members of the genus *Urtica. Leucosyke puya* was placed basally within Urticaceae and formed a distinct lineage, while *Cecropia pachystachya* similarly formed a separate clade. *Oreocnide* formed a highly supported, independent monophyletic subclade comprising 9 species, with all interspecific branches receiving bootstrap support (BS) = 100%. Overall, *Elatostema* and *Pilea* formed sister groups at the core of the family Urticaceae. While *Pilea* was recovered as monophyletic, several traditionally classified species were dispersed in other lineages, reflecting ongoing taxonomic reorganization.

### 3.4 Ancestral state reconstruction of chloroplast genome characteristics

**Figure 3:**
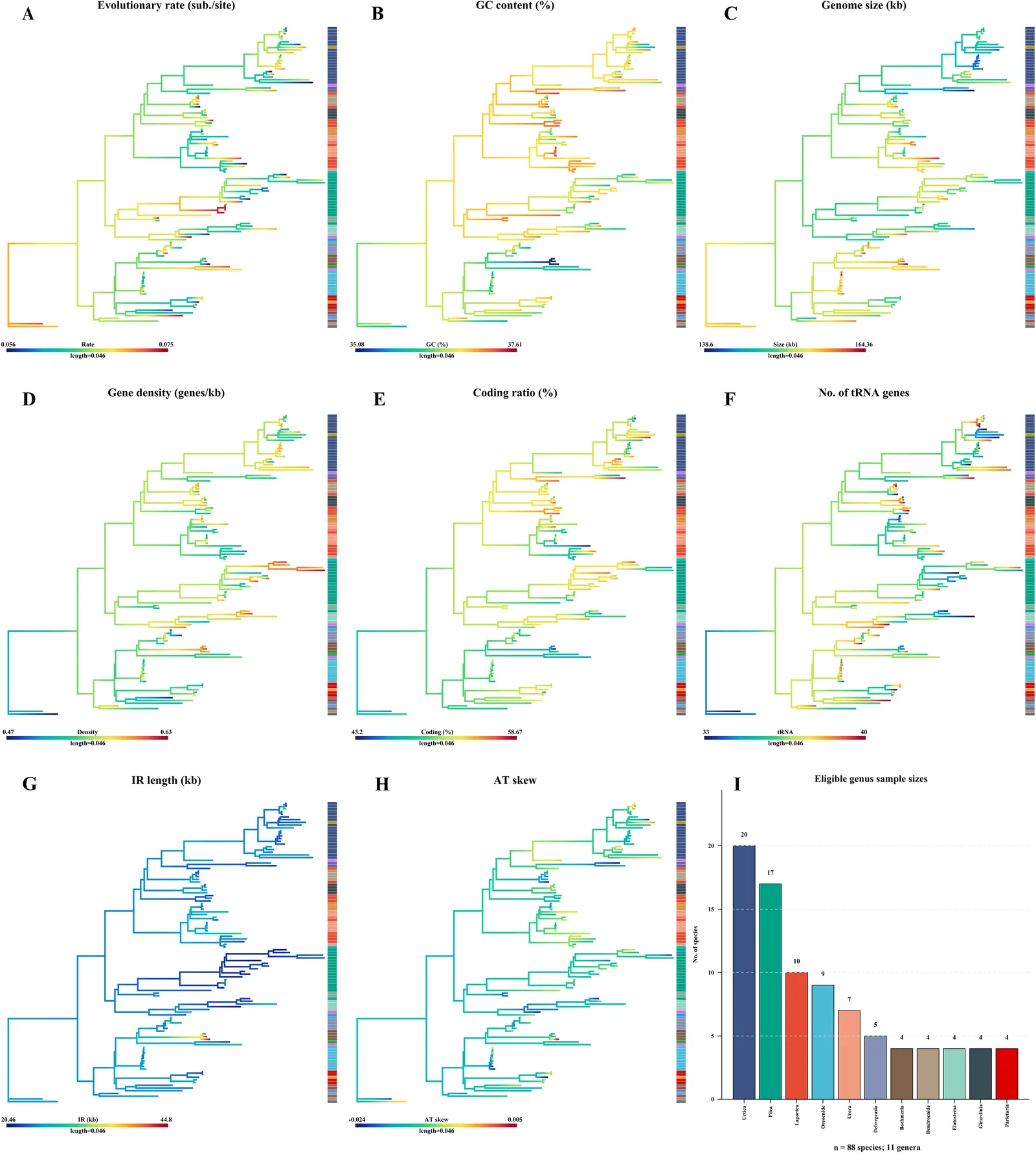
Ancestral state reconstruction of chloroplast genome characteristics. Panels **(A–H)** depict ancestral state transitions mapped onto the Urticaceae phylogeny. **(A)** Evolutionary rate (substitutions per site). **(B)** GC content (%). **(C)** Genome size (kb). **(D)** Gene density (genes per kb). **(E)** Coding ratio (%). **(F)** Number of tRNA genes. **(G)** Inverted repeat (IR) length (kb). **(H)** AT skew. **(I)** Sample size of each genus included in ancestral state reconstruction analysis.

Ancestral state reconstruction was conducted alongside phylogenetic analysis to visualize the evolutionary history of chloroplast genome characteristics within Urticaceae (**Figure 3**). *Elatostema* exhibited consistently elevated evolutionary rates, potentially reflecting lineage-specific evolutionary histories; however, this interpretation requires further testing. In contrast, *Debregeasia* showed comparatively slower rates, potentially suggesting more stable ecological conditions and predominant purifying selection. GC content varied moderately among genera. *Nanocnide* exhibited a relatively high GC content, whereas *Boehmeria* had a low GC content. Overall, GC content remained relatively stable, reflecting conserved selective constraints on nucleotide composition. *Boehmeria* showed higher gene density and gene numbers, whereas *Urtica* exhibited smaller genomes. *Boehmeria* showed the highest coefficient of variation, suggesting greater variation in plastome size among sampled *Boehmeria* species. The genus *Urtica* also displayed marked differentiation in tRNA gene numbers. Notable variation was observed in IR region length among *Boehmeria* and other genera. These regions may undergo frequent expansion, contraction, or rearrangements, making them active sites of chloroplast structural evolution. AT skew values of most taxa were maintained within a near-zero range, demonstrating overall evolutionary conservation of base composition skew. Most lineages retained a nearly neutral base balance over long-term evolution.

### 3.5 Statistical analysis of chloroplast genome variations

**Figure 4:**
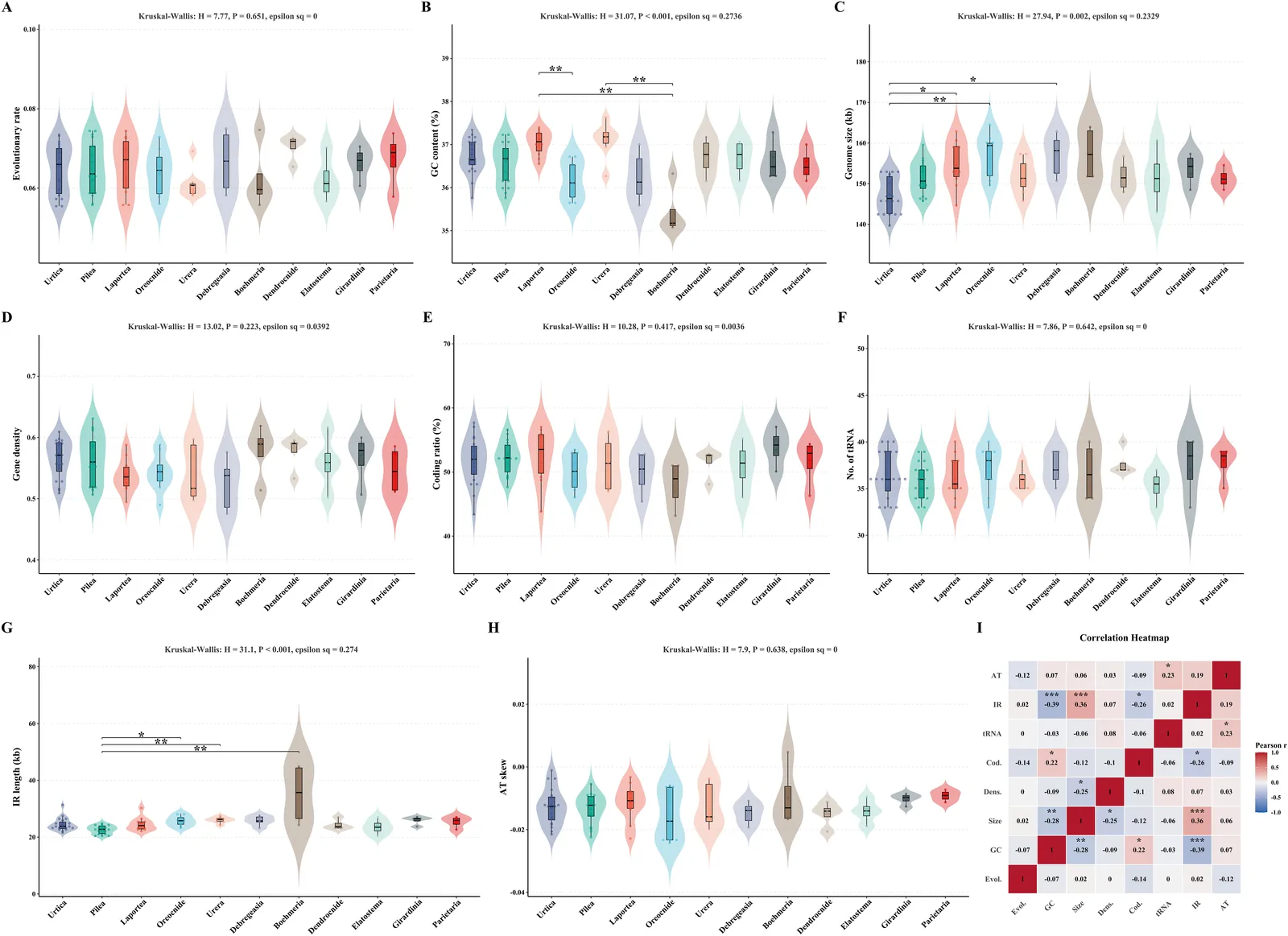
Statistical comparison of chloroplast genome characteristics and correlation analysis. **(A–H)** Distribution of eight genomic features across Urticaceae genera, visualized via violin plots overlaid with box plots. **(A)** Evolutionary rate (substitutions per site). **(B)** GC content (%). **(C)** Genome size (kb). **(D)** Gene density (genes per kb). **(E)** Coding ratio (%). **(F)** Number of tRNA genes. **(G)** Inverted repeat (IR) length (kb). **(H)** AT skew. **(I)** Pearson correlation heatmap among genomic variables. Genera are color-coded by tribe affiliation. The violin width represents the kernel density estimation of the data distribution. Violin plots show the probability density of data. Boxplots display median (horizontal line), interquartile range (IQR; box), and 1.5 × IQR (whiskers). Individual data points are shown as colored dots (beeswarm distribution). Significance levels: *P < 0.05, **P < 0.01, ***P < 0.001; NS, not significant. n = 88 species from 11 genera with >3 species each. In panel I, red indicates positive correlation, blue indicates negative correlation, and the intensity of the color reflects the strength of the correlation.

Chloroplast genome characteristics were compared statistically across 11 genera of Urticaceae (**Figure 4**). Evolutionary rates showed moderate variation among lineages. The Kruskal-Wallis test showed no significant intergeneric difference in evolutionary rate (H = 7.77, P = 0.651, *epsilon squared* = 0) (**Figure 4A**). Violin plots of GC content revealed limited divergence in chloroplast base composition among different genera. Statistically significant differences in GC content were detected between multiple genus pairs including *Laportea* and *Boehmeria*. Among all genera, *Boehmeria* possessed the lowest median GC content across the family, while *Urera* and *Laportea* exhibited relatively high GC proportions (**Figure 4B**). Significant differences in chloroplast genome length were detected among multiple genera including *Urtica*, *Pilea* and *Oreocnide*. Genomes of *Urtica* were generally compact, and stable lineage-specific differentiation in total plastid length was observed across genera (**Figure 4C**). Gene density, coding ratio, tRNA gene count and AT skew exhibited merely marginal or non-significant disparities among genera, revealing greater conservation of these traits relative to other examined plastid characteristics (**Figures 4D–F, H**). IR length varied significantly among genera and remained a major determinant of genome size variation (**Figure 4G**). AT skew values across all genera ranged from -0.02 to 0.02, with most values being positive. This indicates a consistent T > A bias across Urticaceae chloroplast genomes (**Figure 4H**). Pearson correlation analysis revealed significant co-variation among genomic features. IR length was positively correlated with genome size (r = 0.36, P < 0.001) and negatively correlated with GC content (r = -0.39, P < 0.01). This suggests that IR length was associated with both genome size and base composition. Additionally, IR length showed negative correlations with codon coding ratio, implying that structural variation may influence functional elements and codon usage strategies (**Figure 4I**). AT skew showed no significant correlation with other metrics, reflecting its high conservation in the genome. Overall, structural variation (IR region length) was associated with shaping the evolutionary characteristics of Urticaceae chloroplast genomes, co-evolving with functional traits such as base composition and codon usage.

### 3.6 Collinearity analysis

**Figure 5:**
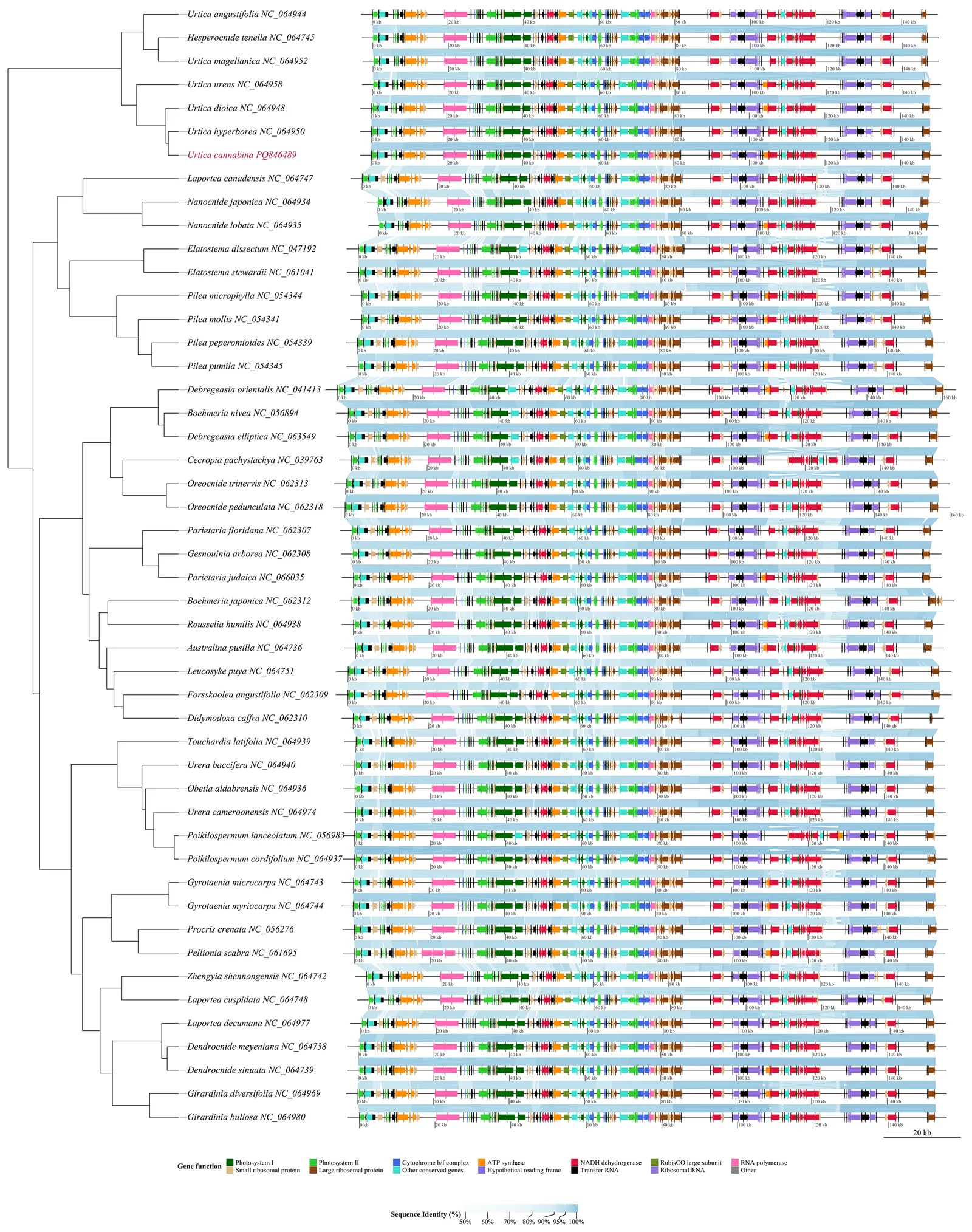
Synteny analysis of chloroplast genomes from 48 Urticaceae species. Linear chloroplast genome maps are arranged according to the clustering tree shown on the left. Collinearity ribbons between adjacent chloroplast genomes are shaded by pairwise nucleotide identity using a pastel sky-blue gradient from 50% to 100%.

Collinearity analysis of chloroplast genomes from 48 Urticaceae species showed that all plastomes retain a conserved quadripartite structure comprising an LSC region, an SSC region, and two IR regions (IRa and IRb). Genome sizes were generally around 150 kb, with highly conserved gene order and content. The newly assembled plastome of *U. cannabina* displayed nearly complete collinearity with other members of tribe Urticeae, especially with *U. dioica*, *U. hyperborea*, *U. urens*, and *U. magellanica*. Gene content and arrangement were identical across these species, and no lineage-specific rearrangements were observed. The close structural similarity among *Urtica* species is consistent with their short phylogenetic branch lengths and indicates limited plastome sequence and structural divergence. Although classified as a distinct genus, *H. tenella* clustered within the Urticeae lineage and showed almost identical genome organization to species of *Urtica*. No obvious differences in gene order or IR boundaries were detected. The high degree of collinearity between *Hesperocnide* and *Urtica* is consistent with the phylogenetic analysis indicating a close evolutionary relationship between the two genera (**Figure 5**; **Supplementary Figure 1**). *H. tenella* harbors an intact *rps19* gene entirely within the IRb region, whereas rps19 is absent from *U. cannabina*. The *ndhF* and *ycf1* genes span the SSC/IR boundaries, with merely a 25–26 bp overlapping spacer; these segments are highly conserved and only carry minor length variations. The intergenic spacer of *trnH-psbA* exhibits subtle interspecific divergence. Closely related species of *Urtica* share a highly conserved overall IR architecture and identical gene arrangement at all junctions.

### 3.7 Simple sequence repeats

**Figure 6:**
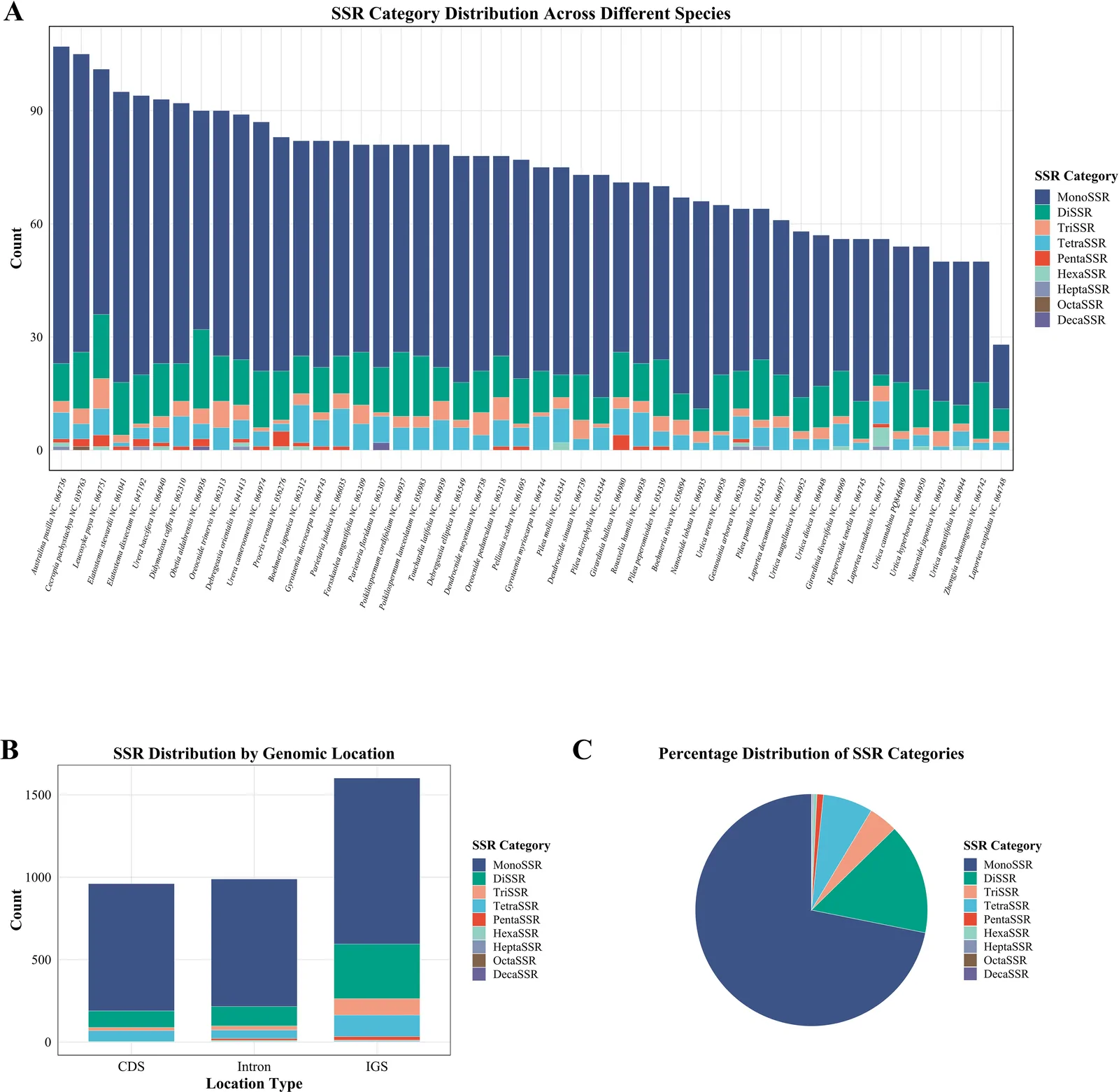
Comprehensive analysis of simple sequence repeats (SSRs). **(A)** Species-level stacked bar plot showing the number and repeat-category composition of SSR loci in each chloroplast genome. Species are ordered by total SSR count, and colors indicate SSR categories defined by repeat-unit length. **(B)** Genomic-location distribution of SSR categories across coding sequences (CDS), introns and intergenic spacer (IGS) regions. Bar height indicates the total number of SSR loci assigned to each sequence context. **(C)** Overall percentage distribution of SSR categories in the complete dataset.

Simple sequence repeats (SSRs) are tandemly repeated DNA sequences and are widely used as molecular markers. SSR distribution was assessed across three genomic compartments of the chloroplast genome: coding sequence (CDS), intron, and intergenic spacer (IGS). Across the 48 Urticaceae species analyzed, SSRs were most abundant in IGS regions (1,602), followed by intron regions (989), and CDS SSRs (961) (**Figure 6B**). Further stratification by genomic location revealed that SSRs of different repeat types were distributed across CDS, intron, and IGS (**Figure 6B**). In terms of repeat type, mononucleotide SSRs (MonoSSRs) were the most prevalent (2,552), followed by dinucleotide (DiSSRs, 550), and tetranucleotide (TetraSSRs, 247) repeats. In contrast, trinucleotide, pentanucleotide, hexanucleotide, heptanucleotide, decanucleotide, and octanucleotide were relatively rare, with counts of 144, 32, 17, 6, 3, and 1, respectively. A stacked bar chart further illustrated the distribution of SSR types among individual species, confirming that mononucleotide repeats were consistently the most abundant SSR class in all Urticaceae chloroplast genomes (**Figure 6A**). Further stratification by genomic location revealed that SSRs of different repeat types were distributed across CDS, intron, and IGS regions, indicating that SSR expansion and retention are not restricted to specific functional elements but occur throughout the chloroplast genome (**Figure 6B**). Mononucleotide SSRs were more abundant than other types. Mononucleotide repeats accounted for 71.85% of all SSRs, followed by dinucleotide (15.48%) and tetranucleotide (6.95%) repeats (**Figure 6C**).

### 3.8 RSCU analysis

**Figure 7:**
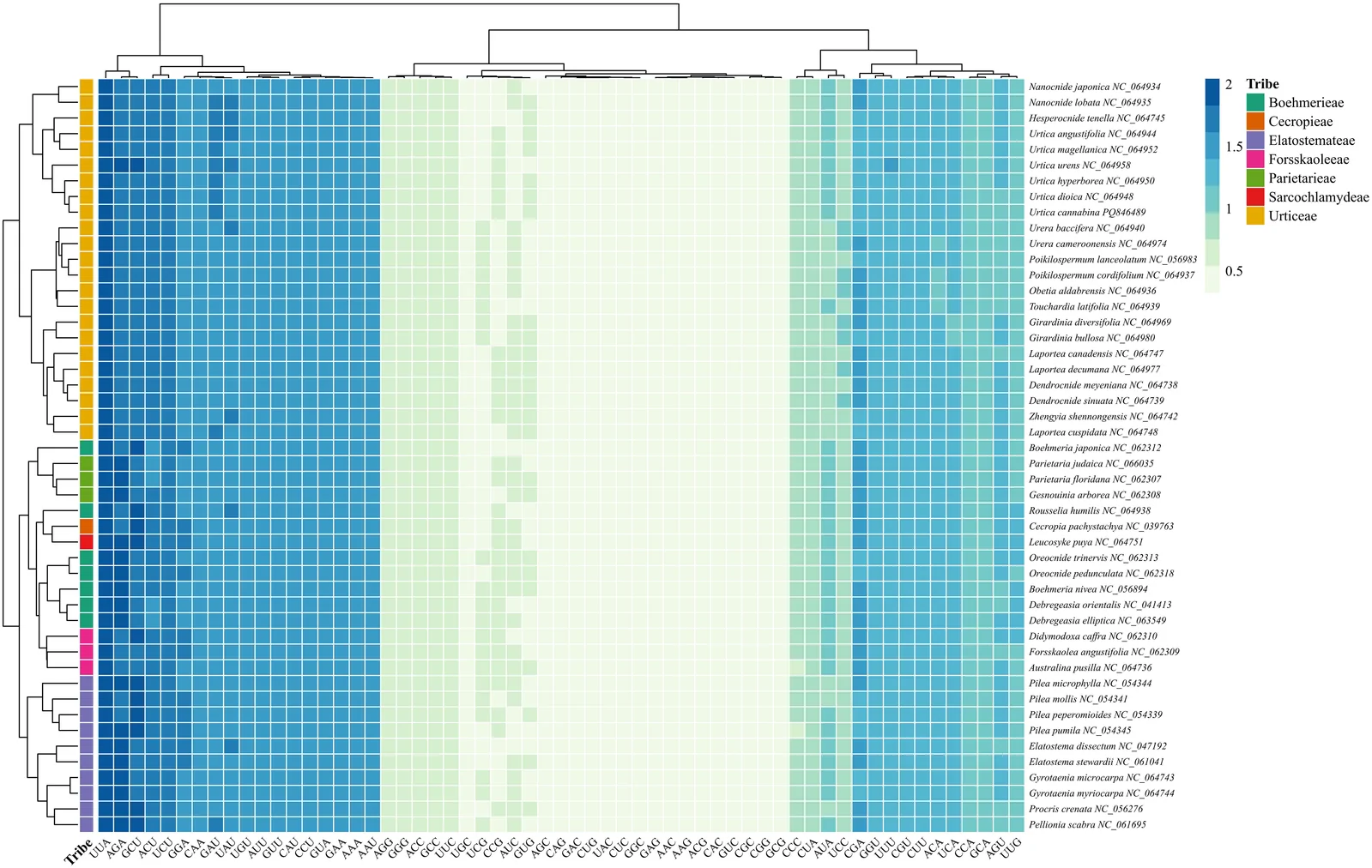
Heatmap of Relative Synonymous Codon Usage (RSCU). Each row represents one species, and each column represents one of the 61 sense codons. Color intensity reflects the degree of codon preference. Species are clustered hierarchically based on overall codon-usage similarity. Left-side annotation bars indicate generic affiliation. GenBank accession numbers are appended to each species label. Scale bar at right indicates RSCU magnitude.

To evaluate the relative contributions of mutational pressure and natural selection to CUB, RSCU values were calculated for protein-coding genes from 48 Urticaceae chloroplast genomes. Most species possessed either 29 or 30 codons with RSCU values greater than 1. Species such as *H. tenella, U. dioica, U. hyperborea, U. magellanica, U. urens, Boehmeria japonica, and U. cannabina* each contained 30 codons with RSCU > 1, whereas the remaining species each contained 29 such codons (**Figure 7**). Fifteen strongly preferred codons (average RSCU > 1.5) were identified, all ending with A/U bases. The most preferred codons were UUA, AGA, and GCU. Notably, leucine codon UUA exhibited the strongest preference across all species. All non-preferred codons with mean RSCU values below 0.5 showed a clear base bias, with terminal bases mainly being C and G.

### 3.9 ENC analysis

**Figure 8:**
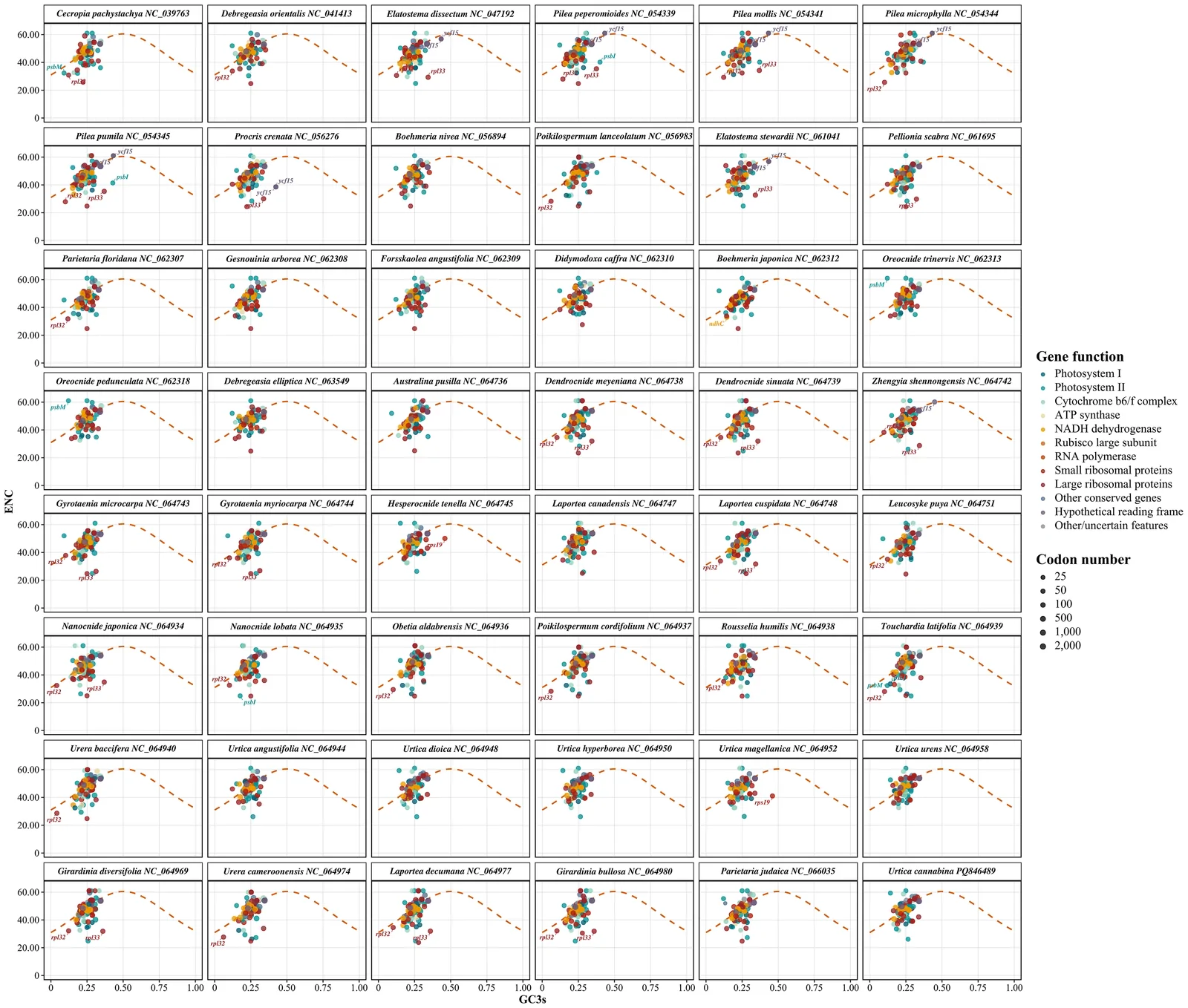
Effective number of codons (ENC) plot analysis. Each panel represents a different species showing the relationship between GC3s and ENC values for individual protein-coding genes. The red dashed line represents the theoretical ENC curve calculated as ENC = 2 + GC3s + 29/[GC3s² + (1 − GC3s)²]. Each point represents a protein-coding gene, colored according to functional category.

ENC-GC3s analysis was conducted on the protein-coding genes of each chloroplast genome across 48 Urticaceae species. The average ENC ranged from 45.59 to 47.22 across species, indicating that overall CUB in chloroplast genes is relatively weak. GC3s values suggested a fairly narrow distribution. The ENC–GC3s plots showed highly consistent distribution patterns among the 48 Urticaceae species. Although slight differences in the degree of deviation were observed among taxa, most genes were distributed below the expected ENC curve, whereas only a small proportion of genes were located on or above the curve (**Figure 8**). Most genes were concentrated within a relatively narrow GC3s range. Photosynthesis-related genes generally displayed relatively high ENC values and clustered in the central region of the plots, indicating comparatively weak codon usage bias. In contrast, several ribosomal protein genes exhibited lower ENC values and a broader distribution pattern.

### 3.10 Parity rule 2 analysis

**Figure 9:**
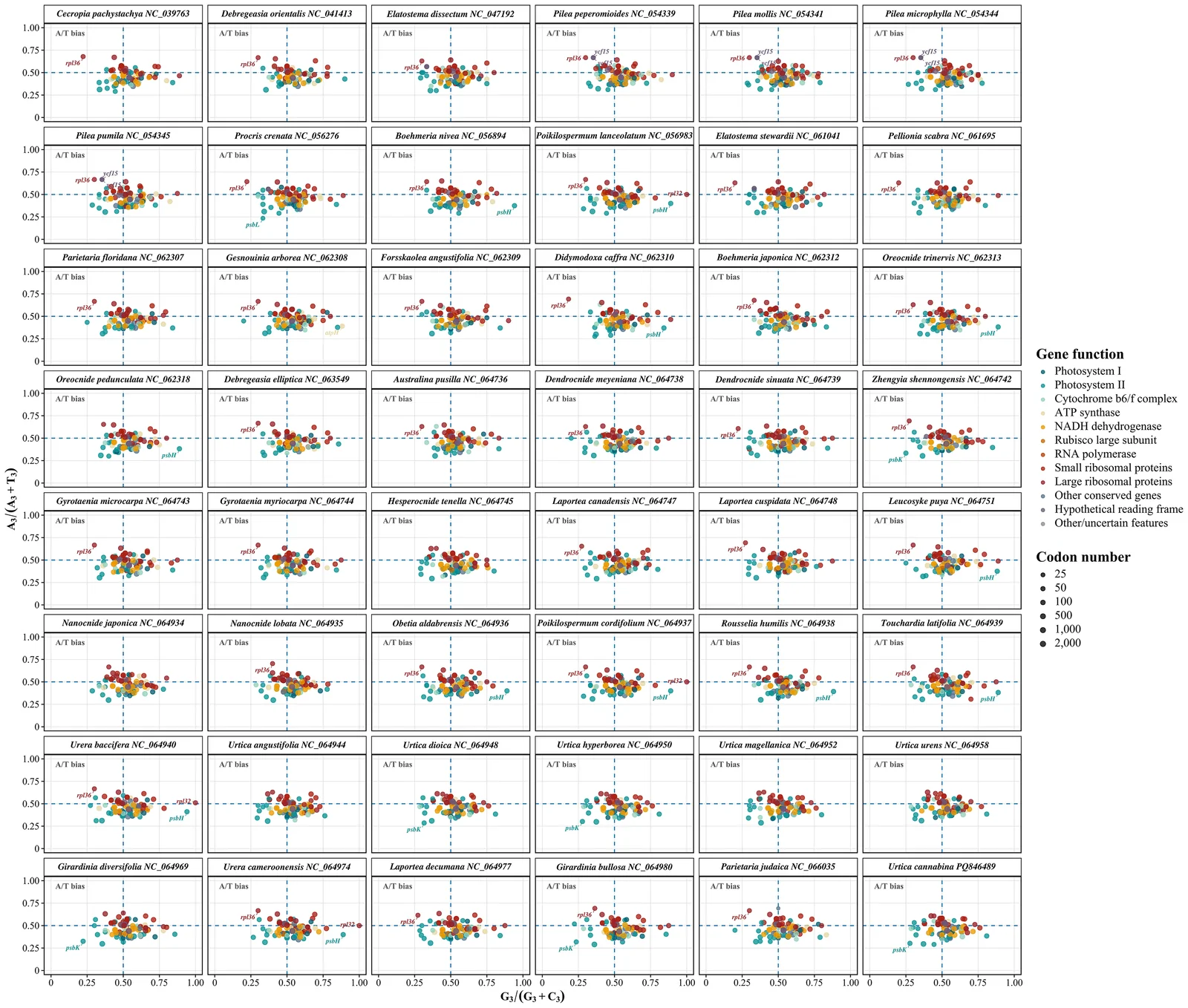
PR2 analysis of codon usage patterns. Parity Rule 2 (PR2) bias plot showing the relationship between G_3_/(G_3_+C_3_) and A_3_/(A_3_+T_3_) ratios at the third codon position for all protein-coding genes. Dashed blue lines indicate the neutral expectation (0.5) under equal frequencies of complementary bases. Deviations from the intersection point (0.5, 0.5) reflect unequal usage of complementary nucleotides.

In all species, genes were unevenly distributed around the theoretical center point (0.5, 0.5), indicating unequal usage frequencies of complementary nucleotides at third codon positions. Most genes were concentrated in the lower-right region of the plots, corresponding to G_3_/(G_3_+C_3_) > 0.5 and A_3_/(A_3_+T_3_) < 0.5. This distribution pattern revealed a general preference for G over C and T over A at synonymous third codon sites. Although the extent of dispersion varied slightly among species, the overall distribution pattern was highly conserved throughout the family. The quadrant analysis further supported this observation (**Figure 9**). Across all 48 species, the G_3_/T_3_-enriched quadrant consistently contained the largest number of genes. On average, 40.67 genes per species were located in this quadrant, accounting for 48.36% of all protein-coding genes. In comparison, the average numbers of genes in the C_3_/A_3_, C_3_/T_3_, and G_3_/A_3_-enriched quadrants were 12.44, 16.19, and 14.81, respectively. The G_3_/T_3_-enriched quadrant represented the dominant category in all 48 species. The proportion of genes located in the G_3_/T_3_-enriched quadrant ranged from 42.35% to 55.17% among species, demonstrating a remarkably consistent nucleotide usage pattern across Urticaceae plastomes. The pronounced accumulation of genes in the G_3_/T_3_-enriched region and the clear displacement from the PR2 equilibrium point indicate widespread nucleotide compositional asymmetry at synonymous third codon positions among Urticaceae chloroplast genomes.

### 3.11 Neutrality analysis

**Figure 10:**
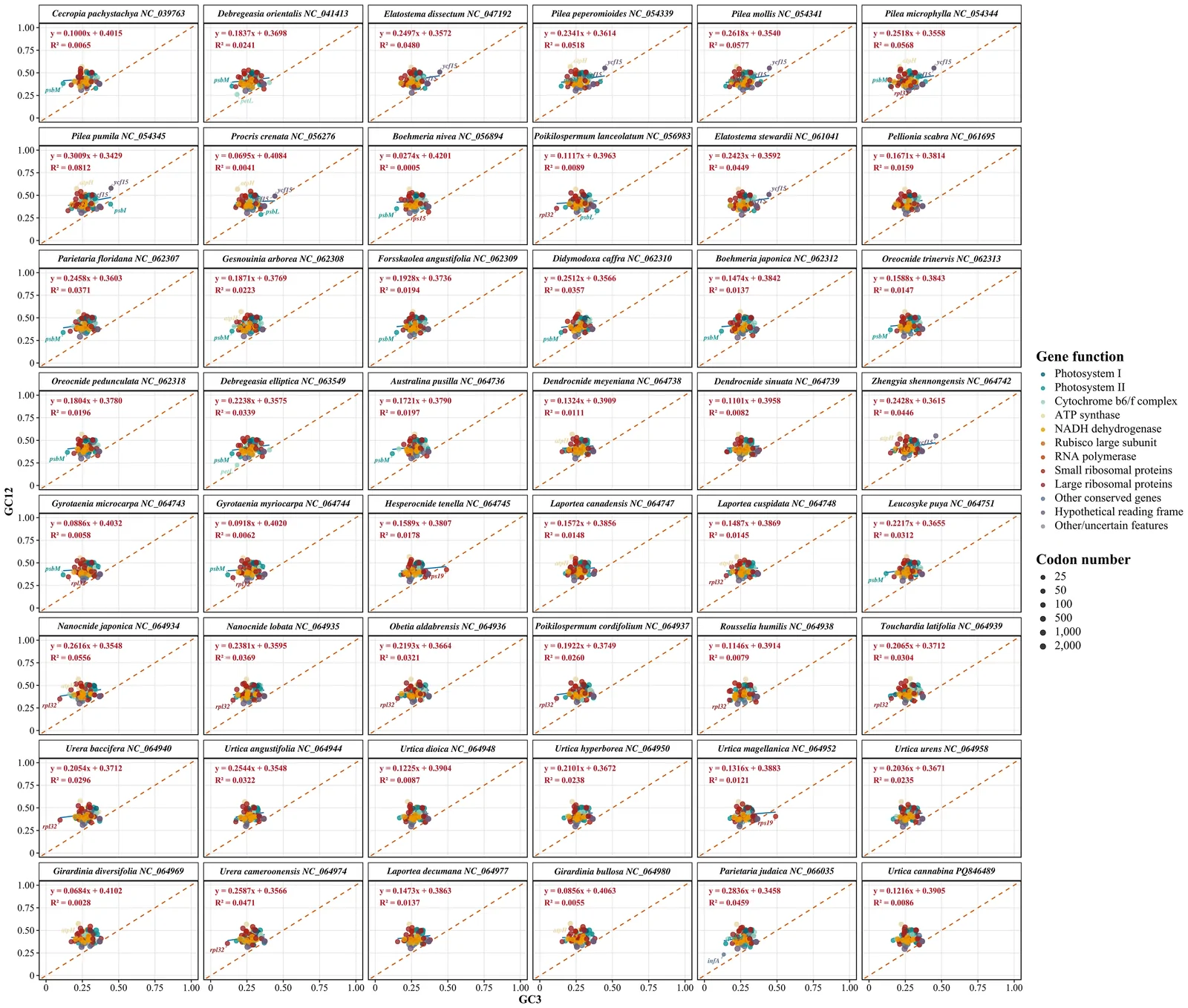
Neutrality analysis of codon usage patterns in Urticaceae chloroplast genomes. Gene function-colored scatter plots showing the relationship between GC content at the third codon position (GC_3_) and GC content at the first and second codon positions (GC_12_) across 48 species from Urticaceae. The red dashed line represents the neutral expectation (y = x). Each panel represents a different species from Urticaceae.

Linear regression analyses revealed positive correlations between GC_12_ and GC_3_ in all species, but the regression slopes were consistently low. The estimated regression slopes ranged from 0.0274 to 0.3009, with a mean value of 0.1799. Most species exhibited slope values below 0.25. The lowest slope was observed in *Boehmeria nivea* (0.0274), whereas the highest value was detected in *Pilea pumila* (0.3009) (**Figure 10**). In addition, the coefficients of determination (R²) were generally low, ranging from 0.0005 to 0.0812, with an average value of 0.0253. Most species displayed R² values below 0.05, indicating weak correlations between GC_12_ and GC_3_. Although slight interspecific variation was observed, the overall distribution pattern was highly conserved throughout the family. Genes were broadly scattered around the regression lines rather than tightly clustered along them, and the low regression slopes were consistently maintained across all taxa.

### 3.12 Nucleotide diversity analysis and selection pressure

**Figure 11:**
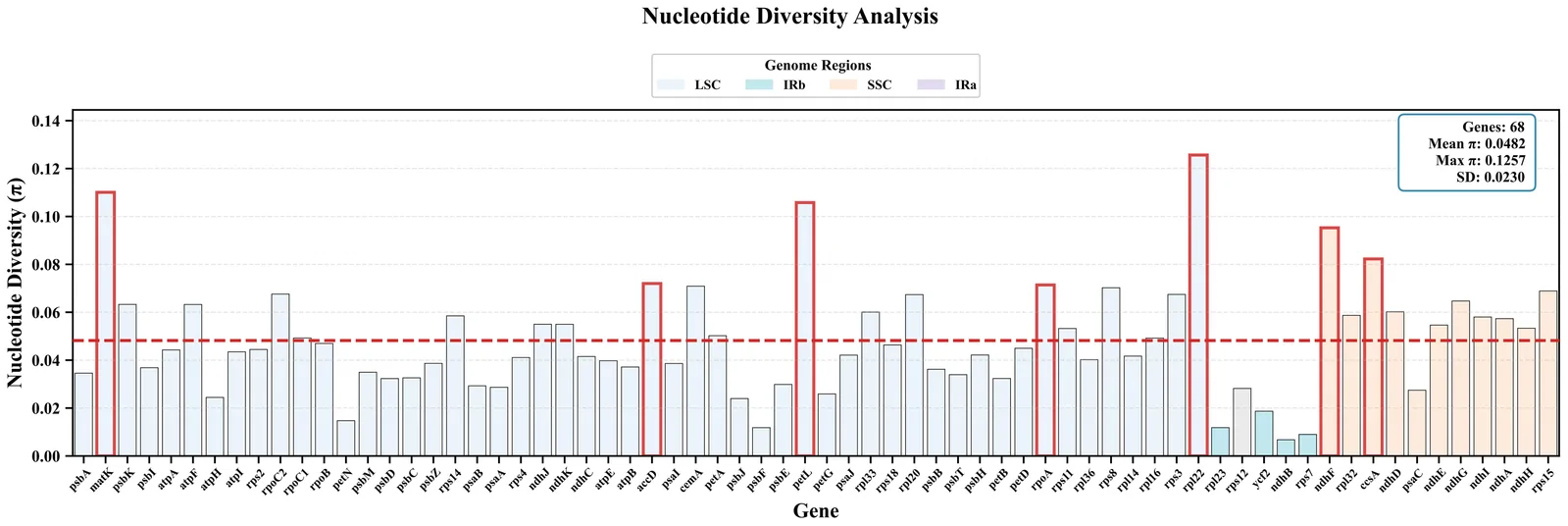
Gene-level nucleotide diversity across Urticaceae chloroplast genomes. The bar plot shows nucleotide diversity (π) for 68 core chloroplast protein-coding genes that passed complete-orthogroup and quality-control filtering. Bars are ordered according to the reference-gene order and colored by chloroplast genome region. The dashed red line indicates the mean π value across the retained genes. The statistics box reports the number of analysed genes, mean π, maximum π and standard deviation.

**Figure 12:**
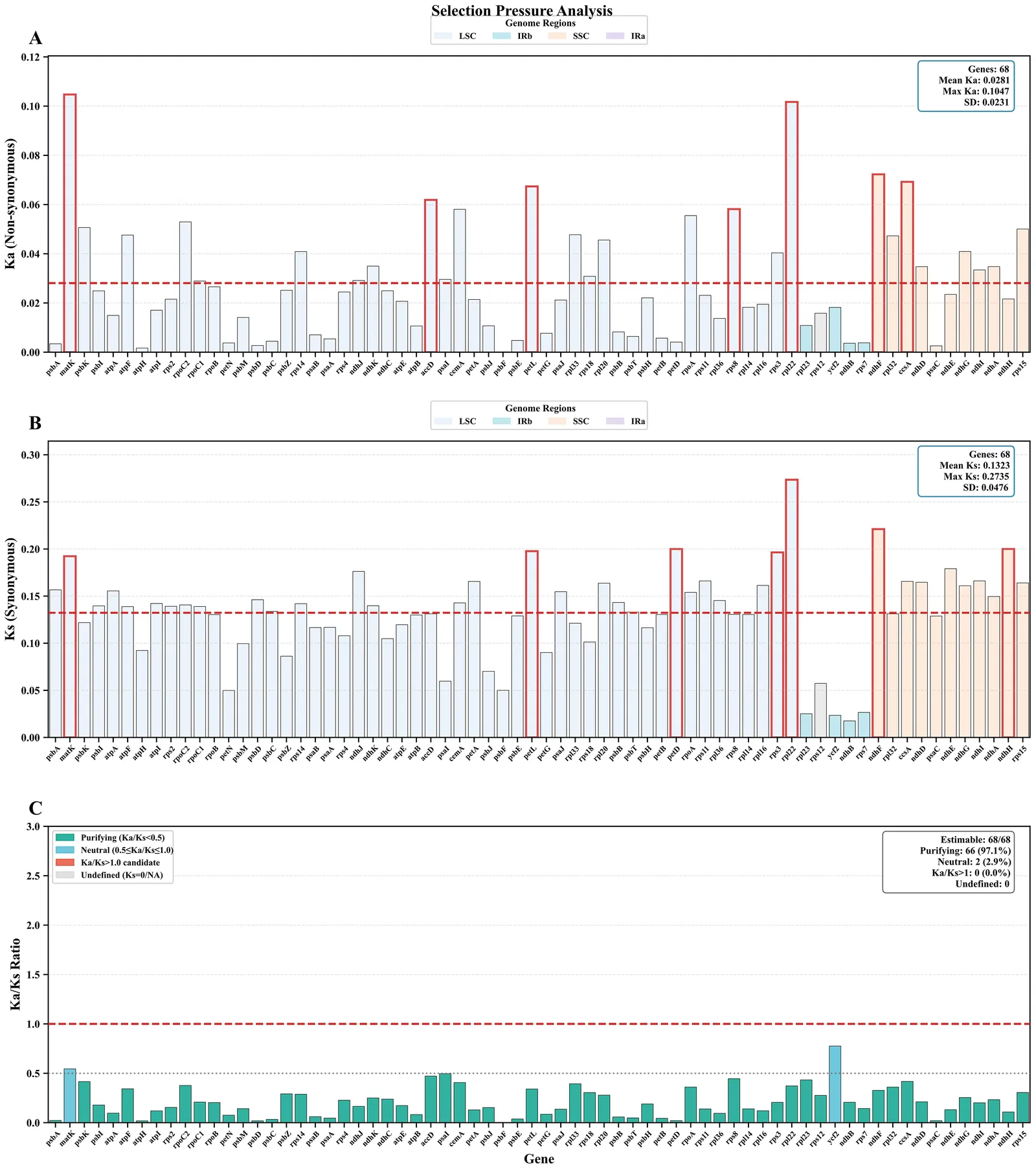
Selection pressure analysis of protein-coding genes in Urticaceae chloroplast genomes. (**A**) Distribution of nonsynonymous substitution rates (Ka) across 68 conserved protein-coding genes. **(B)** Distribution of synonymous substitution rates (Ks). **(C)** Gene-level Ka/Ks ratios calculated from protein-guided codon alignments using the Nei–Gojobori method. Bars are colored according to the selection category: purifying (Ka/Ks < 0.5), neutral-like (0.5 ≤ Ka/Ks ≤ 1.0), Ka/Ks > 1.0 candidate, or undefined. The dashed horizontal lines in panel C indicate Ka/Ks = 0.5 and Ka/Ks = 1.0.

After orthogroup filtering and quality control, 68 core chloroplast protein-coding genes were retained for the main nucleotide-diversity analysis. Comprehensive analysis of 68 protein-coding genes across 48 Urticaceae chloroplast genomes revealed substantial variation in nucleotide diversity. Mean nucleotide diversity (π) across all genes was 0.0482 ± 0.0230, with values ranging from 0.0068 to 0.1257. Nucleotide diversity (π) exhibited distinct variation levels among different functional categories of chloroplast genes. The π value varied substantially between gene functional groups: genes related to protein processing harbored the highest nucleotide diversity (π = 0.0838), followed by RNA polymerase genes (π = 0.0588) and large ribosomal protein genes (π = 0.0568). By contrast, photosystem I (PSI, π = 0.0332) and photosystem II (PSII, π = 0.0347) possessed the lowest sequence variation. These results showed that protein processing-related genes accumulated far more nucleotide substitutions than core photosynthetic complex genes, whereas PSI and PSII coding sequences were highly evolutionarily conserved (**Figure 11**). This indicates moderate, gene-level variation in plastome sequence diversity among the sampled Urticaceae species. The highest π value was observed for *rpl22* (π = 0.1257), followed by *matK*, *petL*, *ndhF*, and *ccsA*, suggesting elevated sequence divergence relative to the genome-wide mean for these genes within the current taxon sampling. In contrast, several genes located in the inverted repeat (IR) regions or belonging to highly conserved photosynthetic/ribosomal gene classes exhibited low π values. The lowest diversity was detected for *ndhB* (π = 0.0068). Notably, the four IRb-region genes retained in the main analysis (*ndhB*, *rps7*, *rpl23*, and *ycf2*) were among the low−diversity genes, consistent with the generally conserved nature of inverted−repeat coding regions. The maximum non-synonymous substitution rate (Ka) was 0.1047, with a mean of 0.0281 ± 0.0231 (**Figure 12A**). The synonymous substitution rate (Ks) ranged from 0.0176 to 0.2735, averaging 0.1323 ± 0.0476 (**Figure 12B**). The ratio of nonsynonymous to synonymous substitution (Ka/Ks) showed that 66 genes (97.1%) exhibited Ka/Ks < 0.5, indicating that nonsynonymous substitutions were generally lower than synonymous substitutions across the chloroplast coding sequences. Two genes, *ycf2* and *matK*, fell within the neutral range, with 0.5 ≤ Ka/Ks ≤ 1.0; no genes showed Ka/Ks ratios > 1.0, indicating that no gene-level signal of positive selection was detected under this analysis. Ka/Ks ratios indicate that strong purifying selection is the dominant evolutionary force (**Figure 12C**).

## 4 Discussion

This study presents a comparative analysis of CUB, selection pressure, and genome structure across 48 Urticaceae plastomes and reconstructs phylogenetic relationships using an expanded 111 species dataset, with *U. cannabina* as the focal species. This work integrates multiple analytical dimensions, including codon usage bias, selective pressure, structural variation and phylogenomics.

### 4.1 Chloroplast genome structure, base composition and codon usage

Chloroplast genomes across Urticaceae exhibit a high degree of structural conservation, with genome sizes ranging from 145 to 160 kb. The structure, gene organization and gene content of the chloroplast genome of Urticaceae species were highly conserved and similar to those of most angiosperms. The *Urtica* chloroplast genome displays a conserved quadripartite organization with stable LSC/IR/SSC boundaries. The IR boundaries were relatively stable, with no evidence of substantial expansion or contraction. Nucleotide diversity was unevenly distributed across the chloroplast genome. The LSC and SSC regions exhibited higher diversity than the IR regions, a pattern consistent with the generally conserved nature of chloroplast genomes. The chloroplast genome exhibits remarkable conservation, a characteristic maintained through various molecular mechanisms including uniparental inheritance, rare occurrence of plastid fusion, and the presence of active repair mechanisms. The IR region is generally considered one of the most conserved regions in the chloroplast genome, and its expansion and contraction have been supported in multiple taxa as important factors driving genome size variation. Abnormal expansion of the IR regions has been observed in taxa such as *Pilea*, *Erodium*, and *Pelargonium*. The expansion and contraction patterns of IR regions across various species in the *Urtica* genus exhibit high similarity, and the distribution and localization of gene types within these regions indicate remarkable consistency. Only minor lineage-specific rearrangements were detected near IR boundaries, consistent with previous findings that boundary variation is one of the few sources of structural diversity in chloroplast genomes (Jansen, 2007; Ulaszewski et al., 2021). In this study, IR length showed a strong positive correlation with genome size and a significant negative correlation with GC content. Collinearity analysis further indicated conservation in gene order and content across all species. This conservation was especially pronounced for photosynthesis-related genes and ATP synthase genes such as *atpA*. Urticaceae chloroplast genomes displayed a pronounced AT-rich bias, a pattern consistent with reports in other plants such as Dipsacales species and wild rice (Asaf et al., 2017; Fan et al., 2018). The decreasing gradients of GC_1_, GC_2_ and GC_3_ further reveal the differences in selective pressures across different codon positions. Given the weak selective pressure acting on the third codon position, GC_3_ is widely adopted as a core parameter for CUB. The ENC-GC3s analysis showed that most genes deviated from the expected curve, indicating that codon usage cannot be explained solely by mutational pressure. PR2 plots revealed asymmetric distributions of nucleotides at synonymous third codon positions, with most genes concentrated in the G_3_/T_3_ quadrant, indicating preferential usage of G over C and T over A. Neutrality analyses further estimated that mutational pressure accounted for only a small proportion of codon usage variation, whereas the majority of the variation was attributable to other evolutionary forces. These results consistently suggest that natural selection represents an important factor shaping codon usage patterns in Urticaceae plastomes. Systematic investigations into plastid codon adaptation have established that translational selection, with *psbA*-type photosystem genes as reference, constitutes the primary driver of codon usage divergence (Suzuki and Morton, 2016).

### 4.2 SSR distribution characteristics

SSRs are typically composed of polyA or polyT repeats and rarely contain G/C repeats, which may be attributed to the higher substitution rates of A/T bases compared to G/C bases (Ye et al., 2018). Mononucleotide SSRs predominated (71.85%), consisting almost exclusively of A/T repeats and mirroring the overall A/T bias of chloroplast genomes (Rasheed et al., 2020; Zhang et al., 2018). Compared with the IR (inverted repeat) region, the higher SSR abundance in the SSC may partially account for its greater variability (Wolfe et al., 1987). Studies have shown that repetitive sequences are closely associated with the majority of insertion/deletion (InDel) mutations, and their abundance is correlated with regions of higher nucleotide diversity (McDonald et al., 2011). In Urticaceae chloroplast genomes, SSRs mainly accumulated in intergenic spacers and introns. Recent comparative analyses consistently indicate that SSR abundance in intergenic spacers and intronic regions mirrors distinct selective pressures; coding sequences are subject to stronger constraints that curb the accumulation of repetitive sequences (Jia et al., 2025; Xiao et al., 2024). These non-coding regions possessed greater nucleotide diversity, suggesting a positive correlation between SSR content and sequence variation. The elevated nucleotide diversity (π) observed in *rpl22 and matK* suggests these loci are substitution hotspots, potentially serving as highly informative markers for lower-level phylogenetics within Urticaceae. The *matK* gene encodes a maturase that is involved in splicing type II introns from RNA transcripts and has been recommended frequently in phylogenetics and barcoding (Hilu et al., 2003). In contrast, genes located in the IR region, such as *ndhB* and *rps7*, showed low diversity, reflecting strong evolutionary constraints. These SSR distribution patterns provide insights into the evolutionary dynamics of Urticaceae chloroplast genomes. Moreover, they facilitate the development of polymorphic chloroplast SSR markers for population genetic studies, species identification, and molecular breeding.

### 4.3 Phylogenetic relationships and collinearity analysis

The phylogenetic tree revealed that *Leucosyke puya* is closely related to *Cecropia pachystachya*. The tribal placement of the genus *Leucosyke* has long been controversial. It was formerly assigned to Cecropieae, while some studies suggested its affinity to Boehmerieae. Consistent with the latest phylogenetic classification, our results support the placement of *Leucosyke puya* within Sarcochlamydeae. The basal placement of Sarcochlamydeae and Cecropieae relative to the remaining tribes suggests an early divergence of these lineages, whereas the more derived positions of Urticeae and Elatostemateae indicate subsequent diversification events. This pattern agrees with previous hypotheses proposing that the evolutionary history of Urticaceae involved successive radiations accompanied by ecological expansion and geographical dispersal (Ogoma et al., 2022; Wu et al., 2013). Species of *Urtica* clustered together and formed a distinct lineage within Urticeae, whereas representatives of *Laportea, Girardinia, Dendrocnide*, and *Poikilospermum* occupied closely related positions within the same tribe. *Pilea, Elatostema, Pellionia, Procris*, and *Gyrotaenia* were grouped within Elatostemateae, consistent with earlier studies based on multilocus and plastome datasets (Monro et al., 2012; Ogoma et al., 2022). Phylogenetic reconstruction based on complete chloroplast genomes revealed that *U. cannabina* clustered closely with *U. dioica* and *U. hyperborea*, forming a strongly supported clade. *U. cannabina* and *H. tenella* exhibited almost complete collinearity with other members of Urticeae. Synteny analyses revealed that *Cecropia pachystachya* and *Leucosyke puya* retained almost identical gene orders, with no major inversions or translocations detected across the family. These observations indicate that plastome evolution in Urticaceae has proceeded in the absence of substantial structural reorganization, consistent with the highly conserved nature of angiosperm plastomes reported previously (Wicke et al., 2011). However, this structural stasis does not preclude substantial nucleotide-level differentiation. A subset of protein-coding genes exhibited elevated nucleotide diversity (π) values. To determine the selective regimes acting upon these variable sites, we conducted Ka/Ks analyses. The vast majority of plastid genes displayed Ka/Ks ratios substantially lower than unity, implying that purifying selection is the dominant evolutionary force, efficiently purging nonsynonymous mutations across the plastome. Together with its short branch lengths in the phylogenetic tree and extensive genomic collinearity with other species of *Urtica*, the lower inferred evolutionary rates may indicate relatively recent divergence and long-term functional conservation. Similar trends were also observed in *H. tenella*, further supporting the close evolutionary relationship with species of *Urtica*. The high degree of collinearity observed between *Hesperocnide* and *Urtica* supports previous molecular evidence indicating a close evolutionary relationship between the two genera *(*Fu et al., 2026*)*. Within individual tribes, most genera were recovered as monophyletic groups with high support values. The prevalence of purifying selection indicates that nonsynonymous substitutions are generally constrained across plastid protein-coding genes. Under this regime, weak CUB may reflect an evolutionary equilibrium where mutational AT bias is partially offset by selection for translational efficiency in a subset of high-expression photosynthetic genes. These results further indicate that chloroplast genomes provide sufficient phylogenetic signal for resolving relationships among major lineages of Urticaceae. Overall, the plastome phylogeny presented here reinforces the revised tribal classification of Urticaceae and provides an evolutionary framework for interpreting patterns of genome evolution and codon usage variation. Broader sampling and ecological modeling will be needed to test whether climatic adaptation has shaped Urticaceae chloroplast genome evolution.

Phylogeny provides a framework that reinforces the tribal classification of Urticaceae. The combination of structural stability and moderate sequence variation renders these genomes ideal markers for both deep and shallow phylogenetic inquiries. While the observed molecular patterns offer a basis for understanding lineage diversification, the adaptive significance of key morphological traits, such as the ecologically consequential stinging hairs characteristic of many Urticeae species, cannot be directly inferred from the present genomic data (Pollard and Briggs, 1982). Future studies combining phylogenomic analyses, comparative morphological assessments, and ecological niche modeling will be essential for evaluating whether the evolution of these defensive traits is associated with lineage-specific adaptations to habitat-dependent herbivory pressures. In addition, fossil-calibrated molecular dating analyses are needed to provide a robust temporal framework for resolving the diversification history and evolutionary trajectories of major lineages within Urticaceae.

## 5 Conclusions

In this study, the complete chloroplast genome of *Urtica cannabina* was assembled and characterized, and comparative analyses were conducted using 48 representative plastomes spanning the major lineages of Urticaceae. Overall, chloroplast genomes within the family exhibited highly conserved quadripartite structures and gene content, whereas moderate variation in genome size and IR boundaries contributed to interspecific differences. Phylogenetic analyses based on protein-coding genes recovered relationships among the major tribes of Urticaceae and were largely consistent with the revised classification framework proposed by recent molecular studies. Sequence divergence analyses indicated heterogeneous levels of nucleotide variability among genes, whereas Ka/Ks ratios below one for most protein-coding genes indicated that plastome evolution in Urticaceae has been dominated by strong purifying selection. Comparative genome analyses further revealed extensive collinearity and the absence of major structural rearrangements, suggesting that chloroplast genome architecture has remained highly conserved throughout lineage diversification. Comprehensive codon usage analyses, including RSCU, ENC-GC3s, PR2, and neutrality plots, consistently suggested that codon usage patterns are shaped predominantly by the interplay between natural selection and AT-biased mutational pressure. The high degree of similarity in codon usage among closely related taxa further suggests the existence of phylogenetic conservatism in plastome evolution. These findings indicate that sequence diversification and codon usage evolution in Urticaceae have proceeded under persistent functional constraints. This study provides valuable genomic resources for *Urtica* and related taxa, enhances our understanding of plastome evolution and codon usage patterns in Urticaceae, and establishes a basis for future investigations of phylogeny, taxonomy, and adaptive evolution within the family.

## Data Availability

The datasets presented in this study can be found in online repositories. The names of the repository/repositories and accession number(s) can be found in the article/**Supplementary Material**.

## Supplementary material

Supplementary Figure 1 Inverted repeat (IR) boundary analysis. This figure illustrates the boundary positions and variation characteristics of the chloroplast genome’s inverted repeats (IRa, IRb) and large single copy (LSC), small single copy (SSC) regions in Urticaceae species. The vertical axis in the figure represents different Urticaceae species, while the horizontal axis represents the length of the genome sequence (kb). Different colored blocks represent the regions of LSC, IRb, SSC, and IRa, respectively. The boundaries between these blocks are the regional boundaries (such as LSC/IRb, IRb/SSC, SSC/IRa, IRa/LSC).

Supplementary Table 1 Genome composition, gene content and codon-usage summary of the analysed chloroplast genomes.
